# Supplementary material for: A peroxisomal ubiquitin ligase complex forms a retrotranslocation channel
Source: Nature. 2022 Jun 29;607(7918):374–80. doi: 10.1038/s41586-022-04903-x (PMC9279156; doi:10.1038/s41586-022-04903-x)

nature portfolio

https://doi.org/10.1038/s41586-022-04903-x

## Supplementary information

# A peroxisomal ubiquitin ligase complex forms a retrotranslocation channel

In the format provided by the authors and unedited

Supplementary Figure1. Uncropped gels and immunoblots

The dash line boxes indicate cropped areas used for each figure.

Fig1b

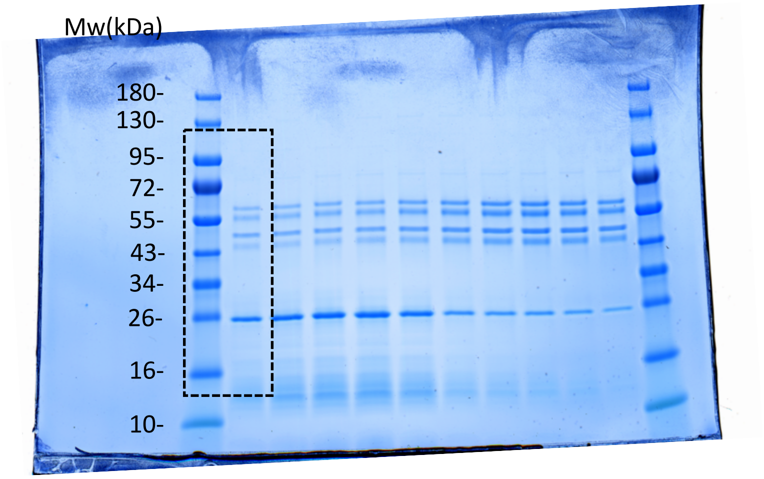


Fig1c

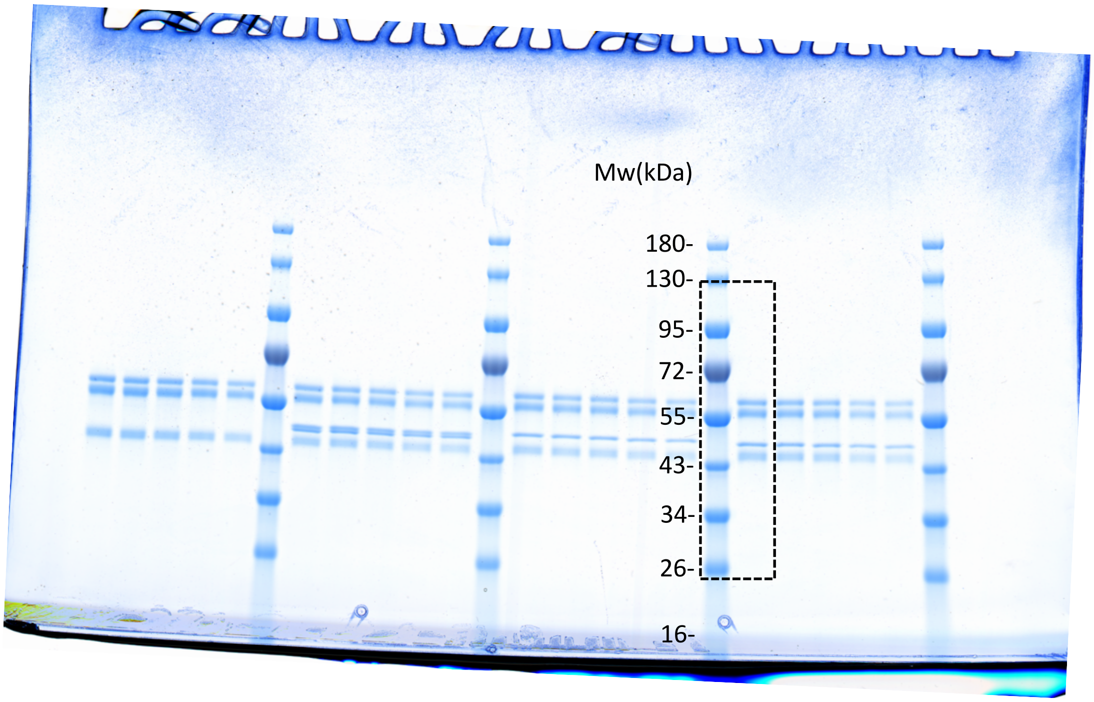

Figure4b

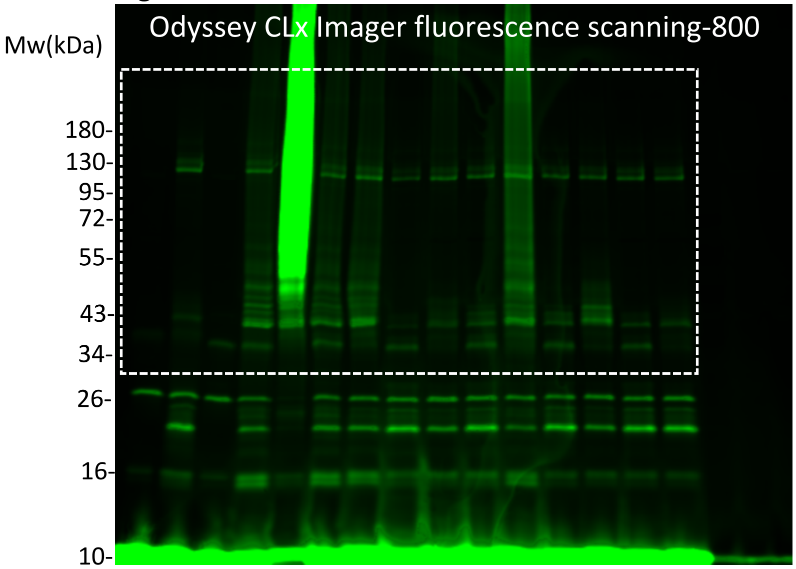


Extended Data Fig2 left panel

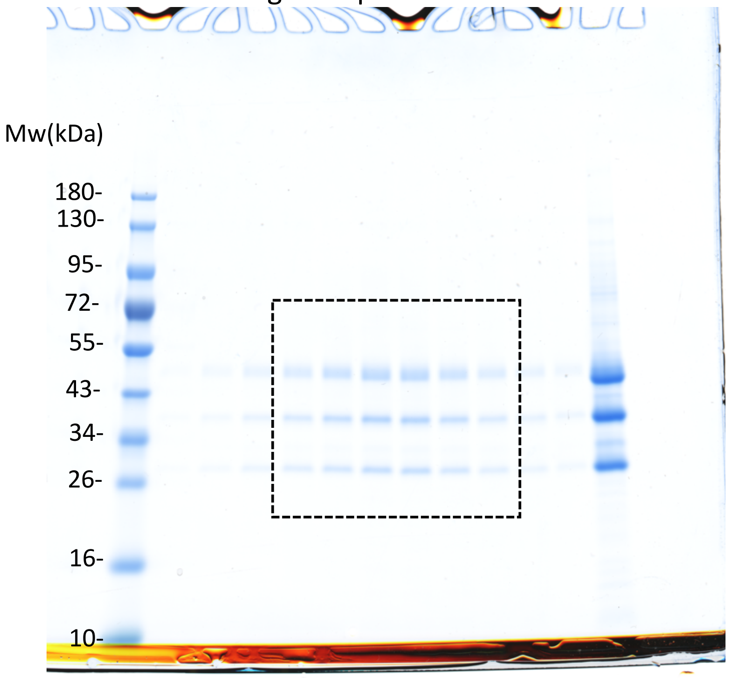

Extended Data Fig2 right panel

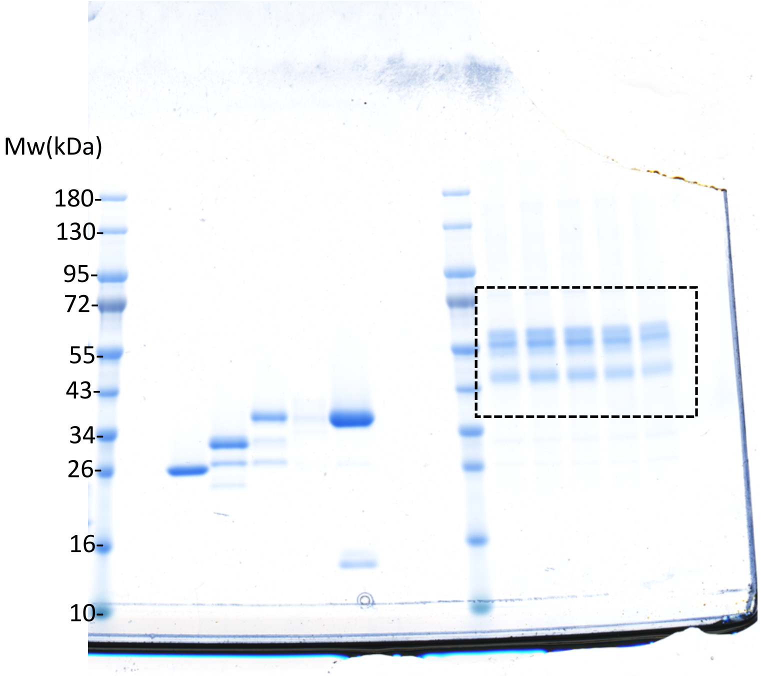


Extended Data Fig6b

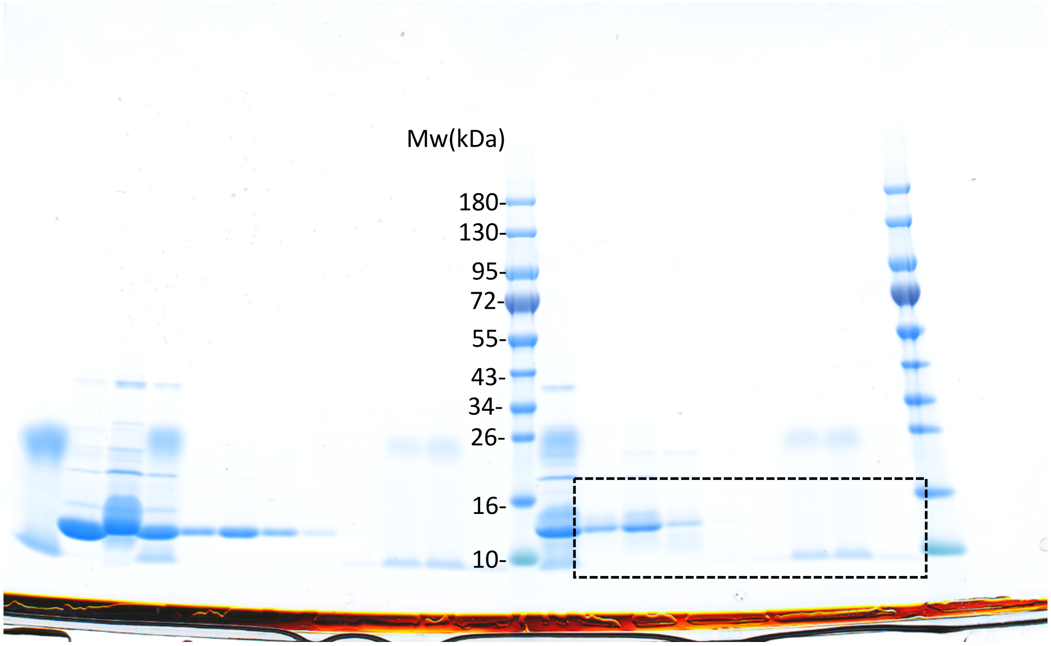

Extended Data Fig6c

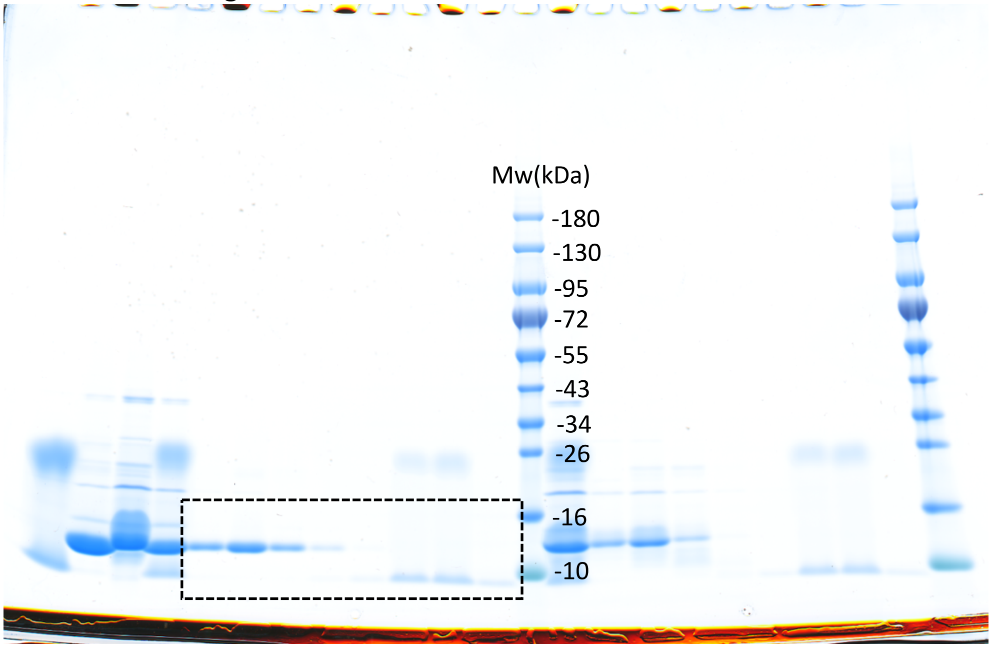


Extended Data Fig6d

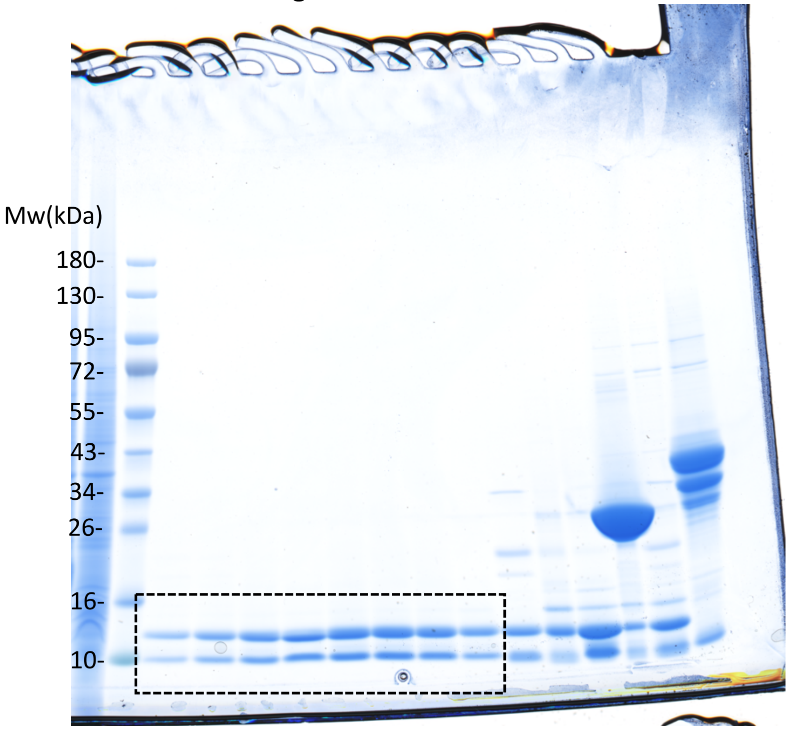

Extended Data Fig6e

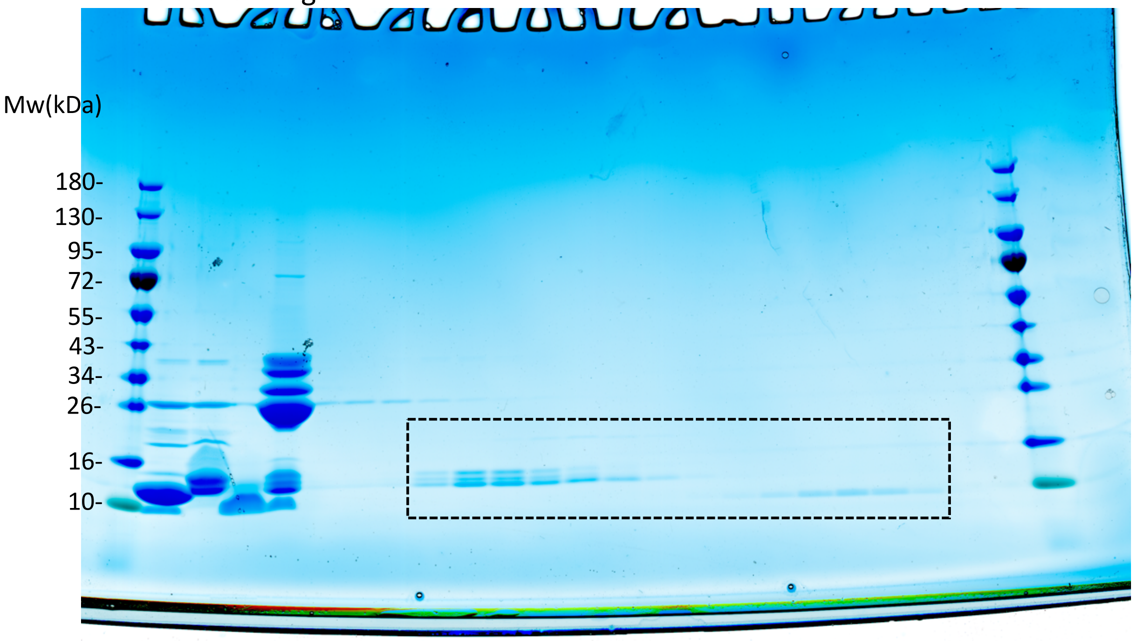


Extended Data Fig6f

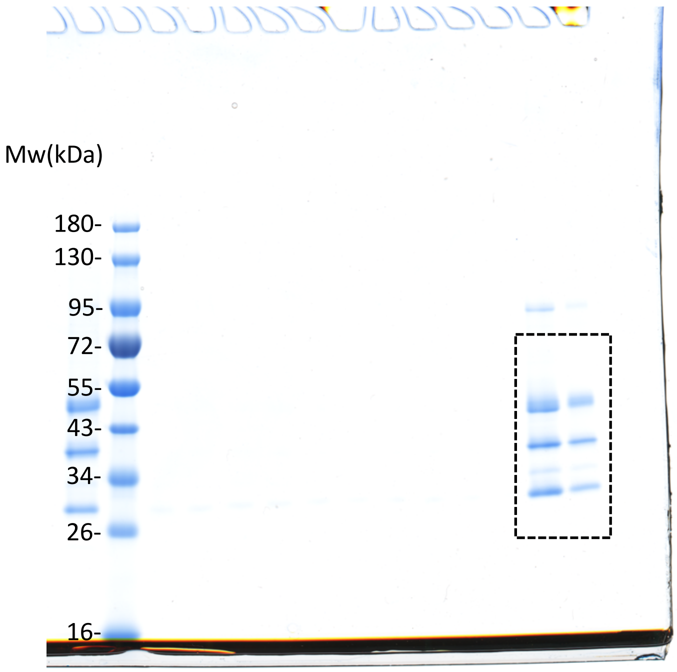

Extended Data Fig6g

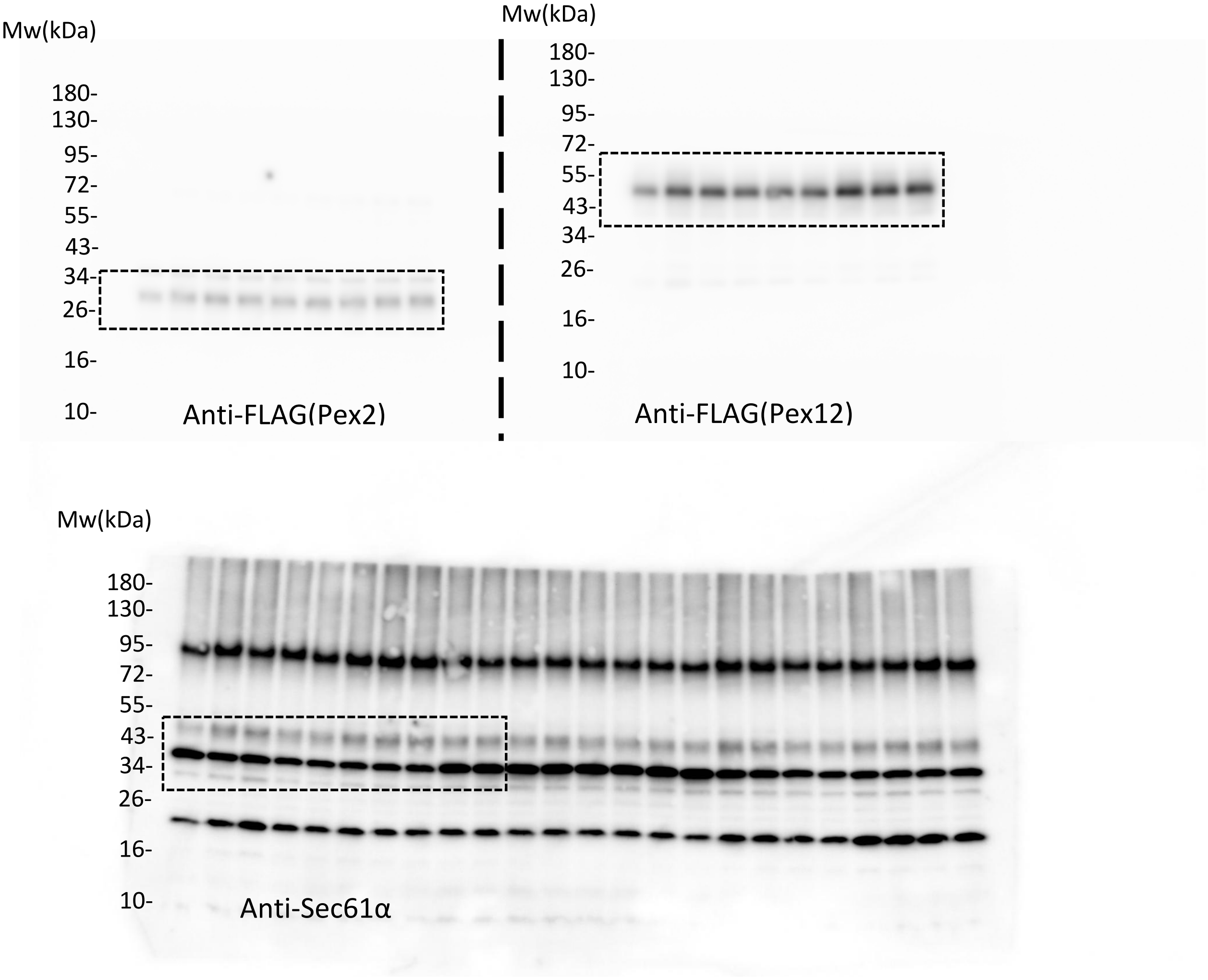

Extended Data Fig10a

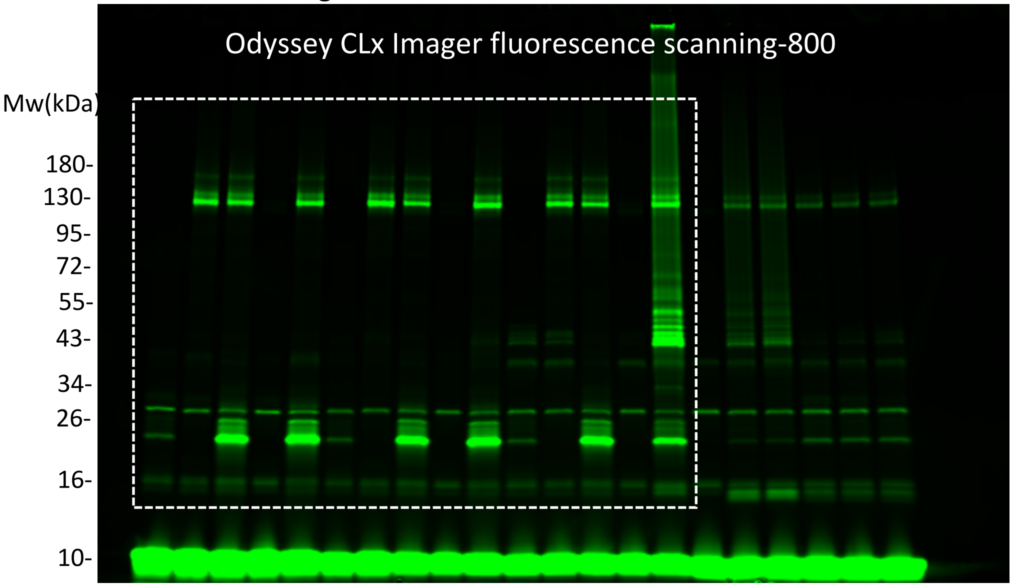


Extended Data Fig10b

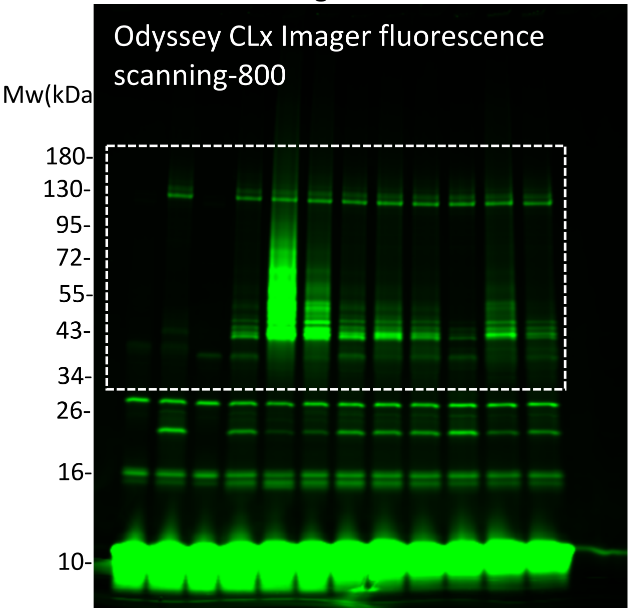

Supplement: Supplementary file 1 — This file contains Supplementary Fig. 1. [file 41586_2022_4903_MOESM1_ESM.pdf]
