# Supplementary material for: A peroxisomal ubiquitin ligase complex forms a retrotranslocation channel
Source: Nature. 2022 Jun 29;607(7918):374–80. doi: 10.1038/s41586-022-04903-x (PMC9279156; doi:10.1038/s41586-022-04903-x)
Supplement: Supplementary file 2 — Reporting Summary [file 41586_2022_4903_MOESM2_ESM.pdf]

nature portfolio

Corresponding author(s): Peiqiang Feng and Tom A. Rapoport

Last updated by author(s): Apr 27, 2022

Reporting Summary

Nature Portfolio wishes to improve the reproducibility of the work that we publish. This form provides structure for consistency and transparency in reporting. For further information on Nature Portfolio policies, see our Editorial Policies and the Editorial Policy Checklist.

Statistics

For all statistical analyses, confirm that the following items are present in the figure legend, table legend, main text, or Methods section.

n/a	Confirmed	
☐	☒	The exact sample size (n) for each experimental group/condition, given as a discrete number and unit of measurement
☐	☒	A statement on whether measurements were taken from distinct samples or whether the same sample was measured repeatedly
☐	☒	The statistical test(s) used AND whether they are one- or two-sided<br>*Only common tests should be described solely by name; describe more complex techniques in the Methods section.*
☒	☐	A description of all covariates tested
☒	☐	A description of any assumptions or corrections, such as tests of normality and adjustment for multiple comparisons
☐	☒	A full description of the statistical parameters including central tendency (e.g. means) or other basic estimates (e.g. regression coefficient) AND variation (e.g. standard deviation) or associated estimates of uncertainty (e.g. confidence intervals)
☐	☒	For null hypothesis testing, the test statistic (e.g. F, t, r) with confidence intervals, effect sizes, degrees of freedom and P value noted<br>*Give P values as exact values whenever suitable.*
☒	☐	For Bayesian analysis, information on the choice of priors and Markov chain Monte Carlo settings
☒	☐	For hierarchical and complex designs, identification of the appropriate level for tests and full reporting of outcomes
☒	☐	Estimates of effect sizes (e.g. Cohen's d, Pearson's r), indicating how they were calculated

Our web collection on statistics for biologists contains articles on many of the points above.

Software and code

Policy information about availability of computer code

Data collection	Cryo-EM data collection was done on a Titan Krios (Thermo Fisher Scientific) electron microscope (300keV) equipped with a Gatan K2 direct electron detector (Gatan). Automatic data collection was done with SerialEM v.3.8-beta. Negative staining EM data collection was done on a Thermo Scientific Tecnai T12 equipped with a Gatan UltraScan 895 (4k x 4k) CCD camera. Crystal data collection was done at APS beamlines 23-ID-C. HKL2000/3000 packages were used for data processing. Quantitative isobaric tag-based proteomics was done on the Orbitrap Lumos Mass Spectrometer (ThermoFisher Scientific) with Proxeon NanoLC-1200 UHPLC (ThermoFisher Scientific) and Accucore Columns (ThermoFisher Scientific).
Data analysis	Cryo-EM and Negative staining EM data processing were done with RELION v.3.1.0, UCSF MotionCor2 (MotionCor2_1.1.0-Cuda80), CTFFIND v.4.1, UCSF Chimera v.1.14, and UCSF ChimeraX v.1.2, PyMOL 2.4.1. Model building was done using Coot 0.9.4, Phenix 1.19.2 and CCP4 7.1.011. Local resolutions were calculated with Resmap v1.1.5. Histograms of directional FSC curves and sphericity values were calculated with the 3DFSC Program Suite Version 3.0. Quantitative isobaric tag-based proteomics data processing was done with MSconvert 3.0 (https://proteowizard.sourceforge.io/tools/msconvert.html) and Comet 2021.02 rev. 0 (http://comet-ms.sourceforge.net/).

For manuscripts utilizing custom algorithms or software that are central to the research but not yet described in published literature, software must be made available to editors and reviewers. We strongly encourage code deposition in a community repository (e.g. GitHub). See the Nature Portfolio guidelines for submitting code & software for further information.

Data

Policy information about availability of data

All manuscripts must include a data availability statement. This statement should provide the following information, where applicable:

- Accession codes, unique identifiers, or web links for publicly available datasets
- A description of any restrictions on data availability
- For clinical datasets or third party data, please ensure that the statement adheres to our policy

The cryo-EM density map and corresponding coordinate of the Thermothelomyces thermophilus Pex2, Pex10, Pex12, Fab complex have been deposited in the Electron Microscopy Data Bank (EMDB) and Protein Data Bank (PDB) under accession codes EMD-25750 and PDB 7T92, respectively. The coordinates and crystallographic structure factors for Saccharomyces cerevisiae RF12 were deposited in the Protein Data Bank (PDB) under the accession code PDB 7T9X. The mass spectrometry proteomics data have been deposited to the ProteomeXchange Consortium via the PRIDE partner repository with the dataset identifier PXD031792 (https://www.ebi.ac.uk/pride/). The structures of the two homodimeric RING domains (RNF4 and BIRC7) bound with corresponding E2~Ub conjugates are under the accession codes PDB 4AP4 and PDB 4AUQ in the Protein Data Bank (PDB), respectively. The structure of GgMFSD2A Fab complex for Fab model building during structure determination is under the accession code PDB 7MJS in the Protein Data Bank (PDB). Uncropped version of all the gels and immunoblot results are included as Supplementary Fig. 1. Source data are provided with this paper.

Field-specific reporting

Please select the one below that is the best fit for your research. If you are not sure, read the appropriate sections before making your selection.

☒ Life sciences ☐ Behavioural & social sciences ☐ Ecological, evolutionary & environmental sciences

For a reference copy of the document with all sections, see nature.com/documents/nr-reporting-summary-flat.pdf

Life sciences study design

All studies must disclose on these points even when the disclosure is negative.

Sample size	No statistical methods were used to predetermine sample size. All functional data were obtained from at least three independent experiments to ensure each data points was repeatable and comparable to other published studies. The amount of proteins for in vitro biochemical experiments was chosen based on previous experience with this specific type of experiments and commonly used sample sizes in the field of research. For single particle cryo-EM reconstruction, sample sizes were determined by available electron microscopy time and the number of particles on each micrograph obtained during the collection time.
Data exclusions	No data were excluded from our analysis.
Replication	The number of replications for each experiment is stated in the Figure Legends.
Randomization	No randomization was performed, since this study did not allocate experimental groups.
Blinding	Blinding is not relevant to this study, as no subjective allocation was involved in any of the structural and functional experiments.

Reporting for specific materials, systems and methods

We require information from authors about some types of materials, experimental systems and methods used in many studies. Here, indicate whether each material, system or method listed is relevant to your study. If you are not sure if a list item applies to your research, read the appropriate section before selecting a response.

Materials & experimental systems

n/a	Involved in the study	
☐	☒	Antibodies
☐	☒	Eukaryotic cell lines
☒	☐	Palaeontology and archaeology
☒	☐	Animals and other organisms
☒	☐	Human research participants
☒	☐	Clinical data
☒	☐	Dual use research of concern

Methods

n/a	Involved in the study	
☒	☐	ChIP-seq
☒	☐	Flow cytometry
☒	☐	MRI-based neuroimaging

Antibodies

Antibodies used	Anti-FLAG antibody produced in rabbit (Sigma #F7425, dilution=1:3000), anti-Sec61 antibody (lab homemade stock, dilution=1:3000).

Validation	The Anti-FLAG antibody from Sigma was validated using immunoblotting as described on the product's website(https://www.sigmaaldrich.com/US/en/product/sigma/f7425). The anti-Sec61 antibody was validated using immunoblotting as described in previous study (Panzner, S., et, al. 1995. Cell).

Eukaryotic cell lines

Policy information about cell lines

Cell line source(s)	Pichia pastoris SMD1168 (Invitrogen-C17500), Saccharomyces cerevisiae UTL7A (gift from Dr. Ralf Erdmann)
Authentication	All cell lines were authenticated with positive control following manufacture's instruction or provided protocol.
Mycoplasma contamination	Mycoplasma contamination is not applicable to this study.
Commonly misidentified lines (See ICLAC register)	We did not use any commonly misidentified cell line.
